# Extracranial Internal Carotid Arterial Dissection With Hypoglossal Nerve Palsy Caused by Cervical Self-Massage: A Case Report

**DOI:** 10.7759/cureus.45516

**Published:** 2023-09-19

**Authors:** Yasushi Shibata

**Affiliations:** 1 Neurosurgery, University of Tsukuba, Tsukuba, JPN; 2 Neurosurgery, Mito Medical Center, Mito, JPN; 3 Neurosurgery, Mito Kyodo General Hospital, Mito, JPN

**Keywords:** acute neck pain, cervical self-massage, hypoglossal nerve palsy, arterial dissection, extra-cranial internal carotid artery

## Abstract

Internal carotid arterial dissection is a relatively rare condition that can cause neck pain and/or ischemic symptoms. Cranial nerve palsy caused by internal carotid arterial dissection is extremely rare. A 39-year-old male massaged his neck with his fingers. He experienced transient neck pain. After one week, he demonstrated hypoglossal nerve palsy. The radiological studies revealed extracranial internal carotid arterial dissection. Cervical self-massage may cause internal carotid arterial dissection and hypoglossal nerve palsy. For the patient with hypoglossal nerve palsy, the extracranial internal carotid artery should be investigated.

## Introduction

There are many causes of lower cranial nerve palsy [1]. Brainstem ischemia and multiple sclerosis are major intra-axial pathology. Cisternal pathologies include schwannoma and meningioma. The lower cranial nerve may be damaged at the skull base foramina or extracranial spaces. Lower cranial nerve palsy caused by vascular lesions is rare. Internal carotid arterial dissection is also a relatively rare condition that can cause neck pain and/or ischemic symptoms [2,3]. Lower cranial nerve palsy caused by internal carotid arterial dissection is extremely rare [4-8]. Here, we report the case of a patient with extracranial internal carotid arterial dissection with hypoglossal nerve palsy caused by cervical self-massage.

## Case presentation

**Figure 1:**
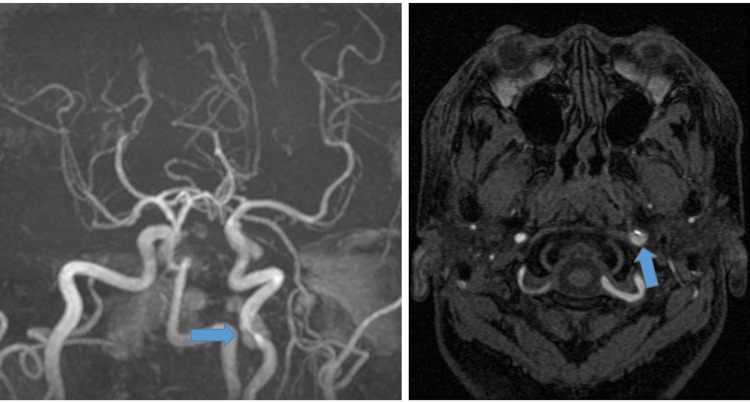
Initial MRA MRA revealed extracranial internal carotid arterial dissection (arrow)

After watching a video on self-massaging on the Internet, a 39-year-old male with no remarkable medical history massaged his own neck with his fingers. He experienced transient neck pain at that time; however, no other symptom was observed at that time. After one week, he experienced dysarthria and difficulty in moving his tongue. Two weeks later, he presented to a local clinic with sustained dysarthria and difficulty in moving his tongue. Magnetic resonance angiography (MRA) revealed extracranial internal carotid arterial dissection (Figure 1).

**Figure 2:**
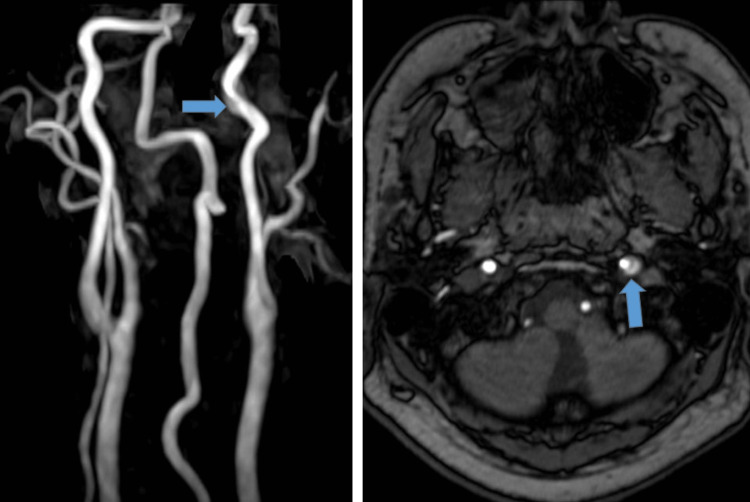
Follow-up MRA Follow-up MRA revealed extracranial internal carotid arterial dissection, which was consistent with the initial MRA finding. Maximum intensity projection image (left) and source image (right)

Diffusion-weighted imaging (DWI) showed no acute ischemic lesions. Therefore, the patient was referred to our hospital. Although alert, he demonstrated mild dysarthria and left hypoglossal nerve palsy. Dysphagia or other neurological abnormalities were not observed. Blood chemistry showed mild hypercholesterolemia. Brain MRI including DWI revealed no abnormality. Follow-up MRA (Figure 2) revealed extracranial internal carotid arterial dissection, which was consistent with the initial MRA finding. His vital signs were stable.

**Figure 3:**
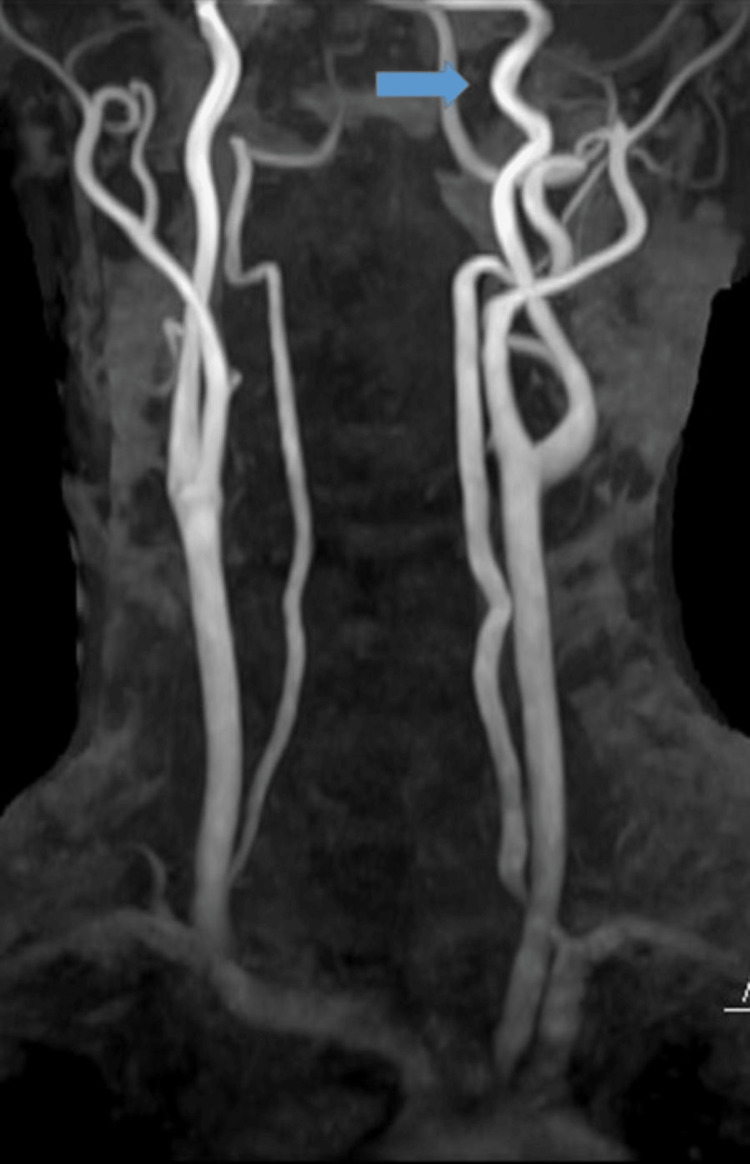
Follow-up MRA after 1.5 months Follow-up MRA after 1.5 months at an outpatient clinic indicated improvement of the dissection

The man was admitted to our hospital and advised bed rest. A carotid ultrasound revealed mild thickening of the intima-media complex; however, carotid dissection around the carotid bifurcation was not observed. Cerebral blood flow imaging using technetium-99m single-photon emission CT also showed no abnormality. Because the patient did not show any ischemic symptoms or findings, we did not prescribe antithrombotic medication. Follow-up MRA one week after admission showed no aggravation, and the patient was discharged. Follow-up MRA after 1.5 months at an outpatient clinic indicated improvement of the dissection (Figure 3).

The patient did not experience any pain and his dysarthria, and his hypoglossal nerve palsy was fully resolved. Ultimately, the patient returned to his normal working life.

We obtained a written informed consent statement of the patient’s approval to report and publish his clinical data in academic journals and meetings.

## Discussion

Cranial nerve palsy associated with internal carotid arterial dissection is frequently observed among men, and the hypoglossal nerve is the most frequently affected cranial nerve by carotid arterial dissection [4-7]. The causes of cranial nerve palsy include direct compression of the dissecting aneurysm or blood flow disturbance in the cranial nerve feeding artery [8]. The inferolateral trunk, often arising from the internal carotid artery, supplies cranial nerves III, IV, and VI and the first division of V. The middle meningeal system supplies cranial nerve VII and the second and third divisions of the trigeminal nerve. The hypoglossal nerve is located near the internal carotid artery, and the feeding artery of the lower cranial nerve is the superior pharyngeal artery branching from the external carotid artery. Thus, generally, the cause of hypoglossal nerve palsy associated with internal carotid artery dissection appears to be the direct compression of the dissecting aneurysm. However, several variations may be encountered in the collateral nutrient arterial supply of the cranial nerves. Because these nutrient arteries are too small to be visualized in routine imaging studies, their lesions are difficult to demonstrate or document [8].

In the present case, the patient himself compressed the extracranial internal carotid artery, and he may have simultaneously compressed his hypoglossal nerve. However, hypoglossal nerve palsy was recognized one week after the compression of the neck. Although there are many prior reports of internal carotid arterial dissection after minor trauma including frequent chiropractor massage [2,9,10], to the best of our knowledge, this is the first report on internal carotid artery dissection caused by a one-time neck self-massage.

Many cases of internal carotid artery dissection involve no cerebral ischemia [4]. In these cases, the dissection site of the internal carotid artery seems to be sub-adventitia. Sub-intimal dissection may be associated with cerebral ischemia. When cerebral catheter angiography cannot identify sub-adventitial dissection [4], MRA and MRI are suitable for demonstrating these dissections without radiation, contrast media, or invasive direct access to the blood vessel.

The optimal treatment strategy for the management of carotid arterial dissection is unknown. However, in cases with no ischemic symptoms but with mild internal carotid arterial stenosis and normal cerebral blood flow, conservative management without antithrombotic medication may be appropriate to avoid complications.

## Conclusions

We report a unique case of extracranial internal carotid arterial dissection with hypoglossal nerve palsy caused by cervical self-massage. Cervical self-massage may cause internal carotid arterial dissection and hypoglossal nerve palsy. For the patient with hypoglossal nerve palsy, even though the patient showed any other neurological or ischemic symptoms, the extracranial internal carotid artery should be considered and investigated.
